# Near Peer Group training on ‘How to be a Foundation doctor’ Course

**DOI:** 10.15694/mep.2017.000124

**Published:** 2017-07-10

**Authors:** Pratik Solanki, Harpreet Singh, Prateek Nalwaya, Humza Mahmood, Safiya Hafeji, Jonathon Dean, Andrew Foster, Lucy Evans

**Affiliations:** 1 Princess Alexandra Hospital NHS Trust

**Keywords:** Undergraduate, Near-peer teaching, Foundation Doctors, Anxiety, Preparedness

## Abstract

This article was migrated. The article was marked as recommended.

Introduction

After graduating from medical school, all UK based doctors enter the Foundation Programme. There is on-going evidence, both anecdotally and published, that final year medical students continue to feel unprepared about starting work. We thus designed a one-day course aiming to improve these students’ preparedness and anxiety levels.

Methods

Pre-course material was provided to the students with information on the skills that were going to be explored in the course. After an initial introduction, there was an interactive demonstration to refresh the students’ knowledge on assessment of an unwell patient using the recognised ABCDE approach- Airway; Breathing; Circulation; Disability; Exposure . Thereafter, the students were split into 10 groups of 3 and 4 and rotated around 10 different stations. Each station was 40 minutes long and breaks were interspersed amongst the teaching to ensure that concentration was maintained. The emphasis was on near-peer teaching with guidance from a recently qualified doctor.

Feedback was requested immediately post-course and three months afterwards.

Results and Feedback

The immediate feedback was very positive with the overall quality rated at 3.93/4.

Regarding the 3 month feedback, there was an average reduction in anxiety levels by 18.3% (p<0.0001) and improvement in perceived preparedness levels by 24.7% (p<0.0001).

All students agreed that the course will help them in preparing to become a foundation doctor and that similar courses should be offered to all final year students.

Conclusions

Practical courses focusing on preparedness can provide a unique opportunity for collaborative training by universities and foundation trusts. These courses are well evaluated and are perceived to improve anxiety and preparedness levels.

## Background

After graduating from medical school, all UK based doctors enter the Foundation Programme, a two year training program with the emphasis to provide them with the supervision and training that they require ( Foundation Programme Reference Guide 2016). It has been recognised that there is a perceived considerable step between being a final year medical student and foundation year 1 (Fy1) doctor ( Kellett et al. 2015), particularly in how well prepared the doctors feel before starting the year. The most recently available General Medical Council (GMC) survey in 2015 suggested that although figures are improving, around 30% of final year students still feel unprepared for their foundation years with the number increasing to 40% at some universities ( GMC National Training Survey 2015). There are several factors that appear to influence this. Van Hamel et al. (2015) suggested that the combination of high levels of stress and anxiety, feelings of discomfort in dealing with critically ill patients and difficulties in prescribing medications all contribute.

There are a number of measures which both foundation trusts and medical schools have implemented to try and improve this situation. After the introduction of *Tomorrow’s Doctors* (GMC 2009) highlighting the importance of student assistantship in the final year of medical school and a ‘shadowing’ period at induction, there has been a large focus on these aspects. Likewise, it is now compulsory for trusts to have an induction program for all Fy1s for every rotation with numerous supervisor meetings ( Foundation Programme Reference Guide 2016). Unfortunately the implementation of these measures appears to be variable, particularly in regards to student assistantship ( Burford et. al 2015) and there remains a large variation in how prepared Fy1s feel according to both medical and foundation school ( Van Hamel et al. 2015). Numerous recent studies have explored these variations between medical schools. Miles et al. (2017) found that those medical schools offering problem-based learning produced more prepared Fy1s. There have been fewer, if any, studies carried out on the variation by Foundation School, but it may involve differences in the induction programme.

The question of what can be done to improve the situation is difficult to answer. Miles et al. (2017) suggested that graduates felt less prepared in areas of paperwork, such as accurate medical records, discharge summaries and death certification and this could be one area to focus on. Likewise, Kellett et al. (2015) also recommended that student assistantship and shadowing, developing clinical reasoning, time management and task prioritising, exposure to acutely unwell patients, practical skills and real life prescribing practice need to be focused on to improve the sense on readiness. In addition, the study carried out by Van Hamel et. al (2015) demonstrated that the perceived most useful aspects of the induction program included Fy1 shadowing, critically ill patient scenarios, prescribing and top tips from foundation trainees. It is likely that that all these different aspects will need to be explored and amalgamate at both medical school and foundation trust level to promote the development of highly skilled and confident foundation trainees ( Miles et al. 2017).

## Introduction to the Course

A number of the issues raised in the background had also been voiced by final year medical students from Barts and the London School of Medicine and Dentistry (BLSMD), an East London based medical school in the UK, whilst on placement at Princess Alexandra Hospital in Harlow. In particular, there was an anxiety about starting work as a Fy1 with a number of concerns including management of acutely unwell patients, prioritisation particularly whilst on-call and paperwork, particularly discharge summaries and death certificates. The decision was thus made by the Medical Education Department to organise a course for these students with the attempt to improve confidence and understanding in these fields. The emphasis was to guide the students through clinical situations and provide them with experiences and an opportunity to reflect. We initially consulted a group of final year students from BLSMD and decided on what learning topics should be concentrated on. The idea was to have small numbers of students rotating though different teaching sessions, giving them an opportunity to experience several aspects of life as a foundation doctor. The emphasis was also on near-peer teaching whenever possible, with the potential advantage of receiving guidance from a junior doctor who had only recently passed their final exams but has the experience of being a qualified doctor, and a number of previous studies and observations have supported this notion ( Rodriques et al. 2009; Rashid et al. 2011).

Initially, we organised a pilot scheme involving the same group of students. We had eight students in total, whom we split into four groups of two and rotated them though four different stations. From the feedback received and further input from the students we created our final list of topics and learning outcomes for each one ( appendix 1). We attempted to use all rungs of the revised Bloom’s Cognitive Taxonomy ( Adams 2015) as relevant with extra emphasis on the application, analysis and evaluation aspects. In addition, we utilised Kolb’s experiential learning cycle ( Yardle et al. 2012) as a basic framework to ensure effective learning was obtained from these active experiences.

## Method

The course was hosted in early 2017. The majority of tutors were current Fy1s and Fy2s (foundation year 2) recruited from various trusts. The tutors were all emailed course packs giving them information and guidance on delivering their session. In total there were 23 tutors for 34 students.

Pre-course material was provided to the students with information on the skills that were going to be explored in the course. After an initial introduction, there was an interactive demonstration to refresh the student’s knowledge on assessment of an unwell patient using the recognised ABCDE approach- Airway; Breathing; Circulation; Disability; Exposure ( Smith et al, 2012; Resuscitation Council UK,2016). Thereafter, the students were split into ten groups of 3 and 4 and rotated around ten different stations. Each station was 40 minutes long and breaks were interspersed amongst the teaching to ensure that concentration was maintained. The ten stations are listed below with a short description of what they entailed:

1. **Mock Ward Round**- A two bedded ward was created with actors as patients and students were expected to take notes and write a job list for the ‘round’. The first patient was a new admission seen on the post-take ward round and the second was a patient ready for discharge. One of the tutors was playing the role of the registrar, presenting the patients’ history, and the other the role of the consultant. All paraphernalia, such as observations charts and drug charts were present by the bed side. Each student had their own set of medical notes for the patients and were documenting within the notes as would occur in real life. Guidance and feedback was then provided on their note taking and an example of good note taking was given to them.
2. **Simulated on-call**- Students were given bleeps and participated in an on-call session. There was an unwell patient simulated by a high fidelity manikin within the simulation suite. Students were bleeped and asked to attend various areas of the building and perform tasks such as assessing an actor with chest pain, prescribing fluids in another ‘ward’ etc. The scenario allowed students to manage an unwell patient during the stressful situation of an on-call with emphasis on prioritising the sick patient.
3. **Surgical emergencies**- interactive case discussions, focussing on diagnosis, treatment and data interpretation. There were two surgical cases in total.
4. **Medical emergencies**- as per surgical emergencies. There were three medical emergency cases.
5. **Prescribing/discharge summaries**-Prescribing scenarios were based on likely situations encountered by Fy1s and prescribing was on real drug charts. A British National Formulary reference book (BNF) was present for each student. The scenarios included warfarin prescription and antibiotics with an emphasis on drug interaction and potential side effects. Discharge summary scenarios were also provided and students were asked to document on paper discharge summaries. The tutors guided them through the process and gave them feedback on their prescribing and discharge summaries.
6. **Practical skills (cannulation/catheterisation)**- Performed in the skills lab on models. There were two cannulation models and two catheterisation models. Students swapped over half way through the session.
7. **Radiology**- Diagnostic radiology session with discussion around the clinical scenarios around the scans, the reasons for performing them and management of the conditions.
8. **Prioritisation/Death certificates**- For the prioritisation session, students were given four tasks to discuss and prioritise. Within the session, three more tasks were added at various times and students discussed how they would prioritise the additions. Death certificate scenarios were also provided. One was a straight forward scenario and the other more complicated with discussion about what should be put in each section of the death certificate. Documentation was on copies of real death certificates.
9. **Airway skills/Chest compressions and defibrillation**- These were performed in the resuscitation room with full resuscitation equipment. The airway skills session involved simple airway manoeuvres, airway adjuncts and using the bag valve mask. The group was split in two, with half initially on airway skills and the other half on chest compressions and defibrillation. The groups then swapped over.
10. **Blood loss**- Interactive case involving a patient with a major upper gastro-intestinal bleed. Guidance was given on the massive blood loss policy, reversal agents and blood checking.

Overall, the day started at 8:15am and finished at 5.35pm. Feedback was obtained from all the students immediately at the end and again at three months post-course. At the three month stage we also explored the impact of the course on anxiety and preparedness.

## Results and Feedback

Overall the feedback was very positive - **Feedback scale: 4=Very good; 3=Good; 2=Average; 1=Poor**. We received initial feedback from all 34 students who attended the course. All averages given below are mean averages.

**Overall Quality: 3.93/4**

**Overall Delivery: 3.91/4**

**Overall Timekeeping: 3.97/4**

**100% of students would recommend the course to a colleague**

Table 1 demonstrates the mean averages of scores reported per session. There were a number of positive comments which have been included in appendix 2. These generally portrayed how beneficial the students found the course and what impact they felt it would have on their final exams and starting work as a Fy1. There were a few negative comments and they are also outlined in appendix 2. They generally suggested wanting more time in certain stations.

**Table 1.** Mean averages of scores reported per session

Station	Content (out of 4)	Delivery (out of 4)
**Mock Ward Round**	**3.79**	**3.70**
**Simulated on-call**	**3.91**	**3.91**
**Surgical Emergencies**	**3.79**	**3.76**
**Medical Emergencies**	**3.79**	**3.67**
**Prescribing/Discharge Summary**	**3.64**	**3.61**
**Practical Skills**	**3.76**	**3.79**
**Radiology**	**3.88**	**3.79**
**Prioritisation/Death certificates**	**3.53**	**3.61**
**Airway skills/Chest compressions**	**3.85**	**3.82**
**Blood Loss**	**3.71**	**3.61**

Feedback scale: 4=very good; 3=Good; 2=Average; 1=Poor

Regarding the 3 month feedback, which was requested after the students had completed medical final exams, we received 24 completed questionnaires (71%). The full results are listed in Table 2. Essentially all students either strongly agreed or agreed that the course will help them in preparation to be a Fy1. Over 95% of students either strongly agreed or agreed that the course helped them with medical finals. All the students felt that similar courses should be offered to all final year medical students.

**Table 2.** Results of 3 month questionnaire

	Strongly Agree	Agree	Not Sure	Disagree	Strongly Disagree
**I feel that the course will help me in preparation to be a Fy1?**	**15** (62.5%)	**9** (37.5%)	**0** (0%)	**0** (0%)	**0** (0%)
**I feel that the course helped me with medical finals?**	**10** (41.7%)	**13** (54.2%)	**1** (4.2%)	**0** (0%)	**0** (0%)
**I feel that similar courses should be offered to all final year medical students?**	**23** (95.8%)	**1** (4.2%)	**0** (0%)	**0** (0%)	**0** (0%)

Total number in bold and percentage in brackets

In regards to exploring the impact of the course on anxiety we asked the students to report on scale of 1 to 10, pre and post-course, with 10 being extremely anxious. We did the same for preparedness, with 10 being very prepared. Results are shown in Table 3.

**Table 3.** Mean average anxiety and preparedness levels reported

	Pre-Course	Post-course
**Mean Average Anxiety level**	**7.00**	**5.17**
**Mean Average Preparedness level**	**3.65**	**6.12**

Anxiety Scale: 1=not anxious; 10=extremely anxious; Preparedness scale: 1=not prepared; 10=completely prepared.

Performing a paired T-test on all the values using software from GraphPad demonstrated that both the reduction in anxiety levels by 1.83 (95% confidence interval: 1.26; 2.41) (p<0.0001) and improvement in perceived preparedness levels by 2.47 (95% confidence interval: 1.81; 3.13) (p<0.0001) were both statistically significant.

## Discussion

The overall aim of this course was to provide students with practical experience and information on life as a Fy1, focussing on topics not normally taught at medical school. Indeed, the reason the course was initially created was because of the informal feedback received from final year students from BLSMD regarding their fears about becoming a newly qualified doctor. This is not a new phenomenon and has been noted several times recently ( Kellett et al. 2015; Monrouxe et al. 2014) and although both trusts and medical schools have attempted to address the situation, the main emphasis has been on shadowing and apprenticeships. Beyond this, little appears to be available for final year medical students to help in their preparation to becoming foundation doctors and this highlights the main positive aspect and attraction of such a course. In fact, the course was so popular, that within 2 hours of being publicised to students from BLSMD, all 40 places had been taken up. The 40 students did eventually become 34, with the inevitable cancellations and the unfortunate railway issues that were present on the day, but does highlight the value which medical students see in it.

Another positive aspect of the course was the nature of the sessions. With exception to the initial ABCDE session, all sessions were small group teaching involving at least 2 tutors and a maximum of 4 students. This ensured that the sessions remained highly interactive and this is reflected by the very positive feedback. Likewise, the near-peer teaching environment also appeared to work well in this situation. The tutors were able to impart real life guidance and provide tips based being on their experiences. The variety of teaching styles offered by the course was another advantage, with some sessions being sit-down discussions and others allowing a practical practice run of certain situations, such as the mock ward round and simulated on-call. If fact, the high feedback scores that were obtained in the simulated on-call suggests that medical students are particularly anxious about being on call and the opportunity for a practice run in a simulated environment was greatly appreciated. This is similar to some of the published study findings, particularly Miles et. al (2017) who found that current Fy1s would have liked more on-call experience and more practical exposure to unwell patients. We believe that the simulated scenario helps with both of these aspects. Additionally, Miles et al. (2017) also highlighted that graduates felt unprepared in paperwork and the course enabled students to gain experience in this field.

An additional positive feature was the impact the course had on students’ perceptions. Overall, the course statistically improved levels of both anxiety and preparedness by around 18% and 25% respectively. Likewise, all the students who completed the post-course questionnaire felt that the course helped them in their preparation to become Fy1s, with over 60% of students strongly agreeing with the statement. On a side note, although not the primary aim of the course, over 95% of students felt the course also helped them with medical finals. It is a telling assertion that all students would recommend the course to a colleague and all students felt that similar courses should be offered to all final year medical students.

The main disadvantage to the course was the time constraint. This was the only negative comment that was voiced by some students, who would have liked more time in some of the stations. With the format of the course and the amount of substance to cover, it would be difficult to allocate more time only to certain stations. This could potentially be improved upon by reducing the number of stations or having the course over two days, but this may not be to the overall advantage of the students and their experience. Likewise, in terms of data interpretation, the information was gathered from a small number of students from one medical school, and it may not be appropriate to extrapolate the findings to all final year medical students. Additionally the course is faculty heavy with high workload, requiring the recruitment of a large number of tutors relative to students but this for deemed a necessity to ensure the learning outcomes were met. Using Kolb’s learning cycle the period of reflection and conceptualisation which is essential for effective learning to occur is near impossible to achieve successfully without the experiences and guidance of these tutors ( Yardle et al. 2012).

Currently, there appears to be a disconnect between the preparation training that universities deliver and foundation trusts give to their prospective trainees. Generally, medical schools tend to prepare students, usually through student apprenticeships, up until medical finals and the summer holidays with the foundation trust only getting involved during ‘Preparation for profession practice (PFPP)’ week, with very little overlap between the two. Miles et. al (2017) mentions the need for medical schools, foundation schools and employing trusts to work closely together to ensure students get the right training in preparedness and courses such as this one can help bridge this gap. It will also enable trusts to develop a course specific to their hospitals which would help to address another issue which Miles et. al (2017) mentions with regard to difficulties relating to new unfamiliar procedures and paperwork. The easiest place for such a course to be placed would be during the PFPP week but, especially as trusts know some 6 months in advance who their new foundation trainees will be, there may be an opportunity to run such a course in advance of this. This will also provide a unique opportunity for students to visit their future employing trust in a more relaxed environment, meet their peers, ask any pertinent questions and highlight to the trust who may be particularly anxious and what extra support can be provided for them. Likewise, it would help to tailor the later induction program to address certain fears and anxieties which had been highlighted during the course but not been previously considered. Of course, this would require an agreement between the medical schools and the foundation schools for students to attend the trust on that set day, but by the popularity found when running this course, students may be more than willing to give up their own time to attend particularly if they perceive it to be of great benefit.

In relation to this, we are planning to run the course again during the induction programme for the incoming Fy1s. We are tailoring the course to become more Princess Alexandra centric and removing sessions which maybe of less benefit. The hope is for the course to remain near-peer, with the outgoing Fy1s providing the bulk, if not all, of the training. There will also be an additional question and answer session at the end of the day with an opportunity to ask questions to the current outgoing Fy1s. As the course will be run on a Saturday and thus will be voluntary for the students, it would allow a further opportunity to investigate the impact and compare those who attended the course with those who did not.

## Conclusion

Courses on preparing final year medical students to become Fy1s provide a unique opportunity for collaboration between medical schools and foundation trusts. These courses are well evaluated by the students themselves and are perceived to improve anxiety levels and preparedness. Unfortunately, evidence is lacking on the impact on foundation trainees and further studies are required to evaluate the effectiveness of such courses fully.

## Take Home Messages

• Anxiety and perceived lack of preparedness continues to be an issue for newly qualified doctors
• Courses which provide practical, hands-on experience are very well evaluated and can help in the transition from medical student to doctor
• Courses on preparing final year medical students to become doctors can also provide a unique opportunity for collaboration between medical schools and foundation trusts.

## Notes On Contributors

Dr Pratik Solanki:

Senior Clinical Teaching Fellow; Contributed with organisation of the course, collecting data, background research and writing the article; Based in the Medical Education Department at Princess Alexandra Hospital NHS Trust, Harlow, UK.

Dr Harpreet Singh:

Foundation Year 2 doctor; Contributed with organisation of the course and collecting data; Currently based at the Princess Alexandra Hospital NHS Trust, Harlow, UK.

Dr Prateek Nalwaya:

Foundation Year 1 doctor; Contributed with organisation of the course and collecting data; Currently based at the Princess Alexandra Hospital NHS Trust, Harlow, UK.

Dr Humza Mahmood:

Foundation Year 1 doctor; Contributed with organisation of the course and collecting data; Currently based at the Princess Alexandra Hospital NHS Trust, Harlow, UK.

Dr Saifya Hafeji:

Foundation Year 1 doctor; Contributed with organisation of the course and collecting data; Currently based at the Princess Alexandra Hospital NHS Trust, Harlow, UK.

Dr Jonathon Dean:

Foundation Year 1 doctor; Contributed with organisation of the course and collecting data; Currently based at the Princess Alexandra Hospital NHS Trust, Harlow, UK.

Mr Andrew Foster:

Simulation and Clinical Skills lead; Based in the Medical Education Department at Princess Alexandra Hospital NHS Trust, Harlow, UK; Contributed to organisation of the course and writing of the article.

Dr Lucy Evans:

Associate Dean Undergraduate Education; Consultant Anaesthetist; Based in the Medical Education Department at Princess Alexandra Hospital NHS Trust, Harlow, UK; Contributed to organisation of the course and proof-reading the article.

## Appendices

### Appendix 1 Learning Outcomes for sessions:

**ABCDE demonstration**

• Recall the different components of the ABCDE approach
• Describe the use of each component during assessment of an unwell patient
• Interpret the findings of the assessment and suggest appropriate management

**Simulated on-call**

• Manage the unwell patient using a systematic approach
• Appraise the different jobs handed over during the oncall and prioritise them accordingly
• Construct a plan on how to tackle the different jobs within your team

**Mock Ward Round**

• Interpret the findings on the ward round and document accurately in the patient notes
• Create an accurate job list based on the above findings
• Compose a patient plan based on the consultant’s input
• Appraise and evaluate the notes taken and propose areas of improvement

**Medical/surgical emergencies**

• Recall the different types of emergencies which can present in the acute phase.
• Accurately interpret the clinical scenario and the data provided
• Formulate a differential diagnosis
• Compose an investigation and management plan

**Prescribing skills**

• Recall frequently used medication in common scenarios
• Describe the side effects of the medications and their consequences
• Analyse the clinical scenario and the patient’s other medications and correctly prescribe appropriate medications

**Discharge Summaries**

• Understand the different sections of the discharge summary and what information goes in each section.
• Decide what to write in each section based on the clinical scenario
• With the help of the tutors, reflect on the summary that has been written

**Practical Skills**

• Recall the procedure for the practical skill from the pre-course material
• Apply this knowledge to perform the practical skill
• With the help of the tutor, evaluate your performance and revise your method as appropriate

**Radiology**

• Describe the findings present on the imaging
• Interpret the findings according to clinical context
• Create a management plan based on the imaging and clinical context

**Prioritising skills**

• Appraise the different jobs and decide upon their importance
• Create and justify a the list of jobs in order of priority
• Debate the additional jobs added and decide upon their position in the list

Death certificates

• Describe the different sections of the death certificate and their significance
• Interpret the clinical information and complete the death certificate

**Airway skills**

• Recognise a patient with airway compromise
• Describe the different tools available to improve airway compromise
• Demonstrate simple airway manoeuvres and using airway adjuncts
• Practice using the bag valve mask in a two person approach

**Chest Compressions and defibrillation**

• Recognise a patient with cardiac arrest and commence effective chest compressions
• Apply the defibrillation pads and analyse the cardiac rhythm
• Confirm the rhythm is shockable and deliver an unsynchronised DC shock

**Blood loss**

• Recognise a patient with severe blood loss
• Appraise the clinical situation and determine an appropriate management plan
• List the medications which can promote bleeding and describe their actions
• Understand the role of reversal agents and their appropriate use
• Be able to carry out a check to ensure that the right blood is given to the correct patient
• Understand the need for this check and the consequences of errors

### Appendix 2 Positive Comments

• Best revision day I have even been to.
• Really good. Very useful. Relevant to FY1
• Perfectly run, no criticism, everyone was amazing
• Very good course. Lots of key facts and tips throughout the day.
• A very good course. Great revision practice for finals.
• Excellent day- great revision and useful skills and techniques. Amazing Food. Helpful and useful. Great.
• Excellent day, really appreciated the food! Learnt Lots.
• This was an amazing day. Thank you so much.
• Very useful to practice and prep for finals.
• Very interactive which was great.
• Such a good day- thank you. Really useful- great for finals. All the staff were so helpful!
• Really good
• Excellent day- thanks for putting it on!
• Especially good were the mock ward round and oncall!
• Excellent. Learnt a lot. Highlighted areas of improvement. Highly recommended.
• Thank you so much! It was excellent.
• Really enjoyable. Very useful. Brilliant.
• Time keeping excellent. Really good day- content really useful for FY1 that is not always covered in medical school. Teachers were all excellent with good insight into their own experiences.
• Very useful.
• Excellent course- very well prepared and ran smoothly. All topics very relevant and I felt challenged and I learnt lots. I would pay for this course.
• Thanks very much. Best course I have done.
• Really good practice.
• Absolutely fantastic course. One of the very best I’ve ever been on.
• Very good course- good revision and preparation for Fy1
• Great course. Simulation and CPR were excellent
• I would pay for this course. But please don’t charge. Its been the most useful finals course this year

#### Negative Comments

• Some stations required more time than others. Some weren’t immediately clear what to do from the start
• Needed more time for prescribing/discharge summary station
• Some stations do not need 20 minutes (eg cannulation), probably save time for other stations. Not enough time for feedback and Q&A in some stations (eg. Ward round/mock oncall)
• Might need a bit more time per station
• Some stations would have been better longer eg prescribing
