# Supplementary material for: Near Peer Group training on ‘How to be a Foundation doctor’ Course
Source: MedEdPublish (2016). 2017 Jul 10;6:124. [Version 1] doi: 10.15694/mep.2017.000124 (PMC10885277; doi:10.15694/mep.2017.000124)
Supplement: Peer review history

# Reviewer report: Reviewer response for version 1

**DOI:** 10.21956/mep.19312.r28175

**Published:** 2017-08-27

**Authors:** Susmita Reddy Karri

**Affiliations:** 1 Topiwala national medical college and BYL nair charitable hospital, mumbai. india

This review has been migrated. The reviewer awarded 3 stars out of 5 Beautiful concept. Great idea. The fact that the tutors themselves were FY students, is something that really helped in designing the course. The results were hardly surprising, considering that the course was meticulously planned and designed for students.I would like to see this study happen on a large scale and across various years of undergrad, so that the students have a basic idea of what is in-store for them after they graduate and plan their study accordingly.

Reviewer Expertise:

NA

No decision status is available

# Reviewer report: Reviewer response for version 1

**DOI:** 10.21956/mep.19312.r28173

**Published:** 2017-07-12

**Authors:** Julie Browne

**Affiliations:** 1 Cardiff University School of Medicine

This review has been migrated. The reviewer awarded 2 stars out of 5 Thank you for submitting this manuscript, which I enjoyed reading. It is always a pleasure to read about courses that have been piloted carefully, designed with great care to meet the students’ needs, delivered with enthusiasm and enjoyment, and then evaluated to improve quality for the future. The fact that the tutors were mostly FY1 and 2 doctors was particularly impressive and this was clearly a major factor in the course’s success.I agree that your methods of evaluation were a little basic, and your conclusions, therefore, might have been rather more cautious; a statistical analysis of a relatively small number of course evaluation questionnaires is a fairly blunt tool to explore an extremely complex area such as anxiety and preparedness for practice. Moreover, it’s fairly obvious that students (especially where they have clear anxieties and concerns) will benefit from and enjoy any well-run course that addresses their learning needs, so your findings are hardly surprising. But your paper is a great description of how good peer to peer clinical teaching can be when it’s done well, with engaged students and teachers, lots of practical work, individual student-centred learning, and a great emphasis on patient care and safe practice. I like your ideas for future work and hope to see you build on these as the course runs into the future.

Reviewer Expertise:

NA

No decision status is available

# Reviewer report: Reviewer response for version 1

**DOI:** 10.21956/mep.19312.r28176

**Published:** 2017-07-11

**Authors:** sathyanarayanan varadarajan

**Affiliations:** 1 SRM Medical College, SRM University

This review has been migrated. The reviewer awarded 4 stars out of 5 This novel article is about preparing newly passed out final year medical students for the two year Foundation Programme conducted in UK medical schools. This article is for the medical educators, clinicians and those who are involved in preparing medical students for Internship and foundation programs. The authors designed a one-day course aiming to improve these students’ preparedness and anxiety levels. The overall aim of this course was to provide students with practical experience and information on life. Before starting the course, Pre-course material was provided. After an initial introduction, there was an interactive demonstration done to refresh the student’s knowledge on assessment of an unwell patient using the recognized ABCDE approach. Thereafter, the students were split into ten groups of 3 and 4 and rotated around ten different stations. Each station was 40 minutes long and breaks were interspersed amongst the teaching to ensure that concentration was maintained. Feedback was obtained from all the students immediately at the end and again at three months post-course. Overall the feedback was very positive.The positive features of the course are the newness and novelty, the nature of the sessions, the impact the course had on students’ perceptions. The main disadvantage to the course was the time constraint.The authors conclude that, Practical courses focusing on preparedness can provide a unique opportunity for collaborative training by universities and foundation trusts. These courses are well evaluated by the students themselves and are perceived to improve anxiety levels and preparedness.So overall it’s an interesting article which addresses the need for preparing newly passed medical graduates to face the challenges as a new doctor with high levels of stress and anxiety, feelings of discomfort in dealing with critically ill patients and difficulties in prescribing medications.

Reviewer Expertise:

NA

No decision status is available

# Reviewer report: Reviewer response for version 1

**DOI:** 10.21956/mep.19312.r28174

**Published:** 2017-07-11

**Authors:** James Read

**Affiliations:** 1 Plymouth University Peninsula Schools of Medicine and Dentistry

This review has been migrated. The reviewer awarded 3 stars out of 5 Thank you for submitting an article to the latest themed edition of Med Ed Publish. I read this article with great interest and you have clearly created a well received course which I'm sure must have taken a significant period of time to design and implement.Preparedness for practice at the point of graduation is clearly important and relevant. In the UK there are a wide variety of approaches to medical education at medical school and this means that there are some courses which students report better prepare them for their first day as an F1 doctor compared to others. These factors are often complex and variable and depend on the medical course, the individual and the environment in which they practice as a new doctor.Your article raises a number of important points about areas that medical students feel less prepared for. It is interesting to hear that the feedback you collected prior to the study suggested that 'administrative' tasks were viewed with anxiety. It would have been good to present this collected feedback as part of your study as it would have helped to explain in more detail why you designed the stations in the way that you did.I have some anxieties about your study methodology. In particular, your anxiety and preparedness scales. Asking students / doctors to indicate on a scale on 1 to 10 is certainly one way of indicating these factors, but the reality is that anxiety and preparedness for practice are complex psychological constructs that cannot be accurately measured a scale of 1 to 10. Indeed, there are validated scales that have been used in previous research and it would have been good if you had considered using these scales. Your use of statistical analysis on this small number of responses on non-validated scales should therefore be viewed with this in mind as the p value does not tell the whole story here.There are also very complex factors that influence preparedness and anxiety outside this one day course. It is therefore impossible to understand how much your course impacted on anxiety and how prepared students / doctors felt. Collecting answers from a 'control' group may have helped a little with this as at present you have not adequately considered how other factors (e.g. preparation for finals, shadowing weeks etc.) also impacted on reported anxiety and preparedness levels.Overall this is an interesting, innovative and clearly appreciated study and I hope that the comments on methodology will help to shape any further research you undertake into this course.Thank you again for submitting your paper.

Reviewer Expertise:

NA

No decision status is available
